# Identification of a Two-MicroRNA Signature in Plasma as a Novel Biomarker for Very Early Diagnosis of Breast Cancer

**DOI:** 10.3390/cancers13112848

**Published:** 2021-06-07

**Authors:** Anna Adam-Artigues, Iris Garrido-Cano, Juan Antonio Carbonell-Asins, Ana Lameirinhas, Soraya Simón, Belén Ortega-Morillo, María Teresa Martínez, Cristina Hernando, Vera Constâncio, Octavio Burgues, Begoña Bermejo, Rui Henrique, Ana Lluch, Carmen Jerónimo, Pilar Eroles, Juan Miguel Cejalvo

**Affiliations:** 1 Oncology Department, Biomedical Research Institute INCLIVA, 46010 Valencia, Spain; aadam@incliva.es (A.A.-A.); igarrido@incliva.es (I.G.-C.); jacarbonell@incliva.es (J.A.C.-A.); acatarina@incliva.es (A.L.); siaso@uv.es (S.S.); ortega_bel@gva.es (B.O.-M.); martinez_mtemar@gva.es (M.T.M.); hernando_cri@gva.es (C.H.); octavio.burgues@uv.es (O.B.); bermejo_beg@gva.es (B.B.); 2 Precision Medicine Unit, INCLIVA, 46010 Valencia, Spain; 3 Bioinformatics and Biostatistics Unit, INCLIVA, 46010 Valencia, Spain; 4 Clinical Oncology Department, Hospital Clínico Universitario de Valencia, 46010 Valencia, Spain; lluch_ana@gva.es; 5 Cancer Biology and Epigenetics Group Research Center, Portuguese Oncology Institute of Porto (CI-IPOP), 4200-072 Porto, Portugal; vera.salvado.constancio@ipoporto.min-saude.pt (V.C.); henrique@ipoporto.min-saude.pt (R.H.); carmenjeronimo@ipoporto.min-saude.pt (C.J.); 6 Centro de Investigación Biomédica en Red de Cáncer (CIBERONC), 28029 Madrid, Spain; 7 Department of Pathology, Portuguese Oncology Institute of Porto, 4200-072 Porto, Portugal; 8 Department of Pathology and Molecular Immunology, School of Medical & Biomedical Sciences (ICBAS)-University of Porto, 4200-072 Porto, Portugal; 9 Department of Medicine, Universitat de València, 46010 Valencia, Spain; 10 Department of Physiology, Universitat de València, 46010 Valencia, Spain

**Keywords:** breast cancer, miRNA signature, biomarker, early diagnosis, plasma

## Abstract

**Simple Summary:**

Breast cancer diagnosis at the initial stage of the disease considerably improves prognosis and survival rates. This retrospective study aimed to develop and validate a plasma microRNA signature as a non-invasive biomarker for early-stage breast cancer diagnosis. We confirmed in a testing cohort of 54 BC patients and 89 healthy volunteers the value of a signature based on miR-30b and miR-99a levels in plasma samples for stage I breast cancer detection. Furthermore, our results were blindly validated in a second cohort of 74 breast cancer and 74 healthy samples. The proposed microRNA signature presented high value as a fast, cost-effective, and non-invasive biomarker for early-stage breast cancer detection, which will lead to a better prognosis for breast cancer patients.

**Abstract:**

The early diagnosis of breast cancer is essential to improve patients’ survival rate. In this context, microRNAs have been described as potential diagnostic biomarkers for breast cancer. Particularly, circulating microRNAs have a strong value as non-invasive biomarkers. Herein, we assessed the potential of a microRNA signature based on miR-30b-5p and miR-99a-5p levels in plasma as a diagnostic biomarker for breast cancer. This two-microRNA signature was constructed by Principal Component Analysis and its prognostic value was assessed in a discovery cohort and blindly validated in a second cohort from an independent institution. ROC curve analysis and biomarker performance parameter evaluation demonstrated that our proposed signature presents a high value as a non-invasive biomarker for very early detection of breast cancer. In addition, pathway enrichment analysis identified three of the well-known pathways involved in cancer as targets of the two microRNAs.

## 1. Introduction

Breast cancer (BC) is the most commonly diagnosed cancer in women and the leading cause of cancer-related death in most countries [1]. The early diagnosis of BC is essential to improve outcomes of patients. It has been widely demonstrated that the 5-year relative survival of patients diagnosed at stage I arises 100%. However, only about 44% of BC patients are diagnosed at an initial stage of the disease despite the demonstrated advantages of screening programs [2,3].

Currently, mammography is the standard breast screening procedure, but its efficacy for dense breasts and women under 40 years old is rather limited. In this context, other techniques such as ultrasounds, magnetic resonance imaging (MRI), or positron emission tomography (PET) may be used, but are not widely available as they are expensive techniques and can lead to over-diagnosis due to a lack of specificity in some cases [4,5]. Thus, it is necessary to develop new specific and efficient screening methods for BC.

In the last few decades, microRNAs (miRNAs) have been proposed as important regulators of cellular activity. miRNAs are non-coding RNAs of 21–25 nucleotides that regulate gene expression at different levels and are involved in numerous biological processes. Focusing on cancer, miRNAs are deregulated in tumor tissues, where they may act as oncogenes or tumor suppressors by targeting genes involved in cancer-related processes such as tumor initiation, proliferation, cell death, angiogenesis, or invasion [6,7,8,9,10,11]. Moreover, many authors have already demonstrated that miRNAs may be useful as biomarkers for diagnosis, prognosis, and response to therapies in different types of cancers including BC [7,12,13,14]. Importantly, miRNAs can be detected in biological fluids such as serum, plasma, or whole blood, thus being promising minimally invasive biomarkers [12,15]. 

Several works described miR-30b-5p and miR-99a-5p to be potential non-invasive cancer biomarkers. Particularly, plasma circulating miR-30b-5p has been validated as a biomarker for the prognosis and detection of lung and breast cancer [16,17,18,19]. In addition, plasma miR-99a-5p has been demonstrated to serve as a biomarker for the detection of bladder [20] and breast cancer [21,22], as well as for the prognosis of head and neck squamous cell carcinoma [23] and pancreatic cancer [24].

In the present retrospective study, we sought to identify and to validate a two-miRNA signature based on the detection of miR-30b-5p and miR-99a-5p in plasma using Principal Component Analysis (PCA) to diagnose BC at a very early stage.

## 2. Materials and Methods

### 2.1. Study Design and Sample Collection

This retrospective study enrolled plasma samples from healthy donors and BC patients from two different cohorts from independent institutions. The discovery cohort comprised 89 healthy donors and 54 BC patients from the Portuguese Oncology Institute of Porto (IPO-Porto, Portugal), and the validation cohort included 74 healthy donors and 74 BC patients from Biomedical Research Institute INCLIVA and Valencian Biobanking Network (Spain). Plasma samples were collected in accordance with the Declaration of Helsinki. Peripheral blood was collected in EDTA-containing tubes and centrifuged at 2000 rpm for 10 min at 4 °C. Then, plasma was isolated and stored at −80 °C until further use. All BC plasma samples were collected before any treatment. The ethical committees of IPO-Porto (CES-IPOFG-120/015) and INCLIVA (2019/196) approved this study. Written informed consent was obtained from all participants.

### 2.2. MiRNA Extraction

miRNA extraction from plasma samples was carried out using miRNeasy Serum/Plasma Kit (Qiagen, Hilden, Germany) following the manufacturer’s protocol. RNA was quantified at the NanoDrop spectrophotometer (Thermo Fisher Scientific, Waltham, MA, USA) and stored at −80 °C until further use.

### 2.3. Retrotranscription and Quantitative Real-Time Reverse Transcription Polymerase Chain Reaction (qRT-PCR)

A volume of 9.16 µL of RNA from plasma was retrotranscribed into cDNA using the High-Capacity cDNA Reverse Transcription kit (Thermo Fisher Scientific, Waltham, MA, USA), according to the manufacturer’s protocol. The reaction mixture was incubated at 16 °C for 30 min, at 42 °C for 30 min, and at 85 °C for 5 min in a thermal cycler. To determine miRNA expression levels, qRT-PCR was performed. A total of 2 µL of cDNA was amplified with 5 µL of Xpert Fast Probe 2× MasterMix (GRiSP, Portugal), 0.5 µL of Taqman microRNA assays (Assay ID 000602 and ID 000435 for miR-30b-5p and miR-99a-5p, respectively, Thermo Fisher Scientific, Waltham, MA, USA) and 2.5 µL of nuclease-free water. qRT-PCR reaction was carried out on a QuantStudio 5 Real-Time PCR System (Thermo Fisher Scientific, Waltham, MA, USA) under the following conditions: 98 °C for 3 min, 45 cycles of 95 °C for 10 s, 60 °C for 30 s, and 37 °C for 30 s. RNU38B (assay ID 001004 Thermo Fisher Scientific, Waltham, MA, USA) was employed to normalize the expression of miRNAs. A standard curve of four serial 10-fold dilutions of cDNA was run in each plate and used to calculate the expression of miRNAs. All samples were analyzed in triplicate.

### 2.4. Target Prediction Analysis

To identify relevant targets of miR-30b-5p and miR-99a-5p, the online tool DIANA miRPath-v3.0 was used to perform an *in silico* analysis. This tool allows the identification of predicted miRNA targets as well as the significantly regulated KEGG pathways [25]. The algorithm miRTarBase v7.0 was used to select the validated targets of miRNAs of interest. Threshold score was 0.8.

### 2.5. Statistical Analysis

Differences in miRNA expression between two groups were evaluated by using the Mann–Whitney U non-parametric test. Kruskal–Wallis test was performed to evaluate associations between miRNA expression and clinical variables. The combined signature was calculated using Principal Component Analysis (PCA) in the discovery cohort and applied to the validation cohort. To evaluate whether miRNAs individually or the combined signature had prognostic potential, optimal cut-point was calculated using Youden Index [26]. Receiver Operating Characteristic (ROC) curves were constructed and Area Under the Curves (AUC), sensitivity, specificity, and accuracy were calculated. All statistical analyses were carried out using GraphPad Prism 6.01 (GraphPad Software, La Jolla, CA, USA). Tests with *p*-values < 0.05 were considered statistically significant. 

## 3. Results

### 3.1. Study Workflow

**Figure 1:**
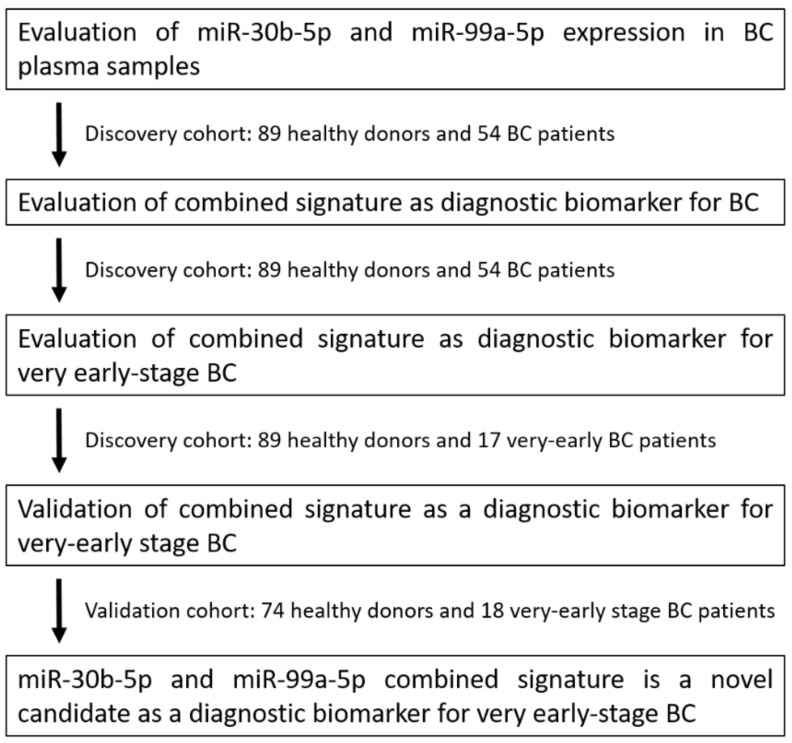
Study design to develop a novel combined miRNA signature biomarker.

Herein, we propose a combined signature of miR-30b-5p and miR-99a-5p levels in plasma as a candidate biomarker to diagnose very early-stage BC; very early stage was defined as TNM stage I [27]. First, miR-30b-5p and miR-99a-5p expression was determined in plasma samples from BC patients and healthy controls. Next, the combined signature was evaluated as a BC diagnostic biomarker and a very early-stage BC diagnostic biomarker in the discovery cohort. Then, the signature was evaluated as a very early BC diagnostic biomarker in the validation cohort (Figure 1).

### 3.2. Circulating miR-30b-5p and miR-99a-5p Levels in Plasma Samples from BC and Healthy Patients

To assess miRNAs’ circulating levels in plasma, a set of 89 samples from healthy donors and 54 plasma samples from BC patients from IPO-Porto (discovery cohort) was selected. Clinicopathological data from the discovery cohort are detailed in Table 1. No correlation was found between patients’ age and miRNA levels for any group.

**Figure 2:**
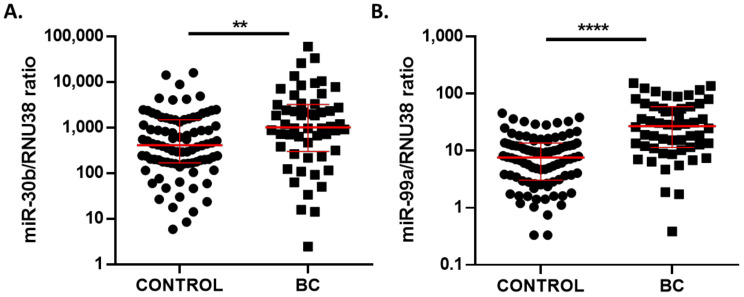
miR-30b-5p (**A**) and miR-99a-5p (**B**) expression levels in plasma samples of 54 BC patients and 89 healthy controls from discovery cohort. Red lines represent median ± interquartile range. ** *p* < 0.01; **** *p* < 0.0001.

Circulating miR-30b-5p levels were significantly higher in samples from BC (median 1012, 95% CI 710.4–2172) compared to healthy controls (median 411.5, 95% CI 242.1–614) (*p* = 0.0023) (Figure 2A). In the same trend, miR-99a-5p levels were significantly higher in BC (median 26.94, 95% CI 15.48–36.14) than in volunteers’ plasma samples (median 7.59, 95% CI 5.44–10.05) (*p* < 0.0001) (Figure 2B). Furthermore, correlation analysis showed that circulating miR-30b-5p and mir-99a-5p levels in plasma presented a positive correlation with Spearman’s correlation coefficient of 0.3677 (*p* < 0.0001). 

### 3.3. Two-MicroRNA Signature as a Potential Diagnostic Biomarker in Very Early-Stage BC Patients

**Figure 3:**
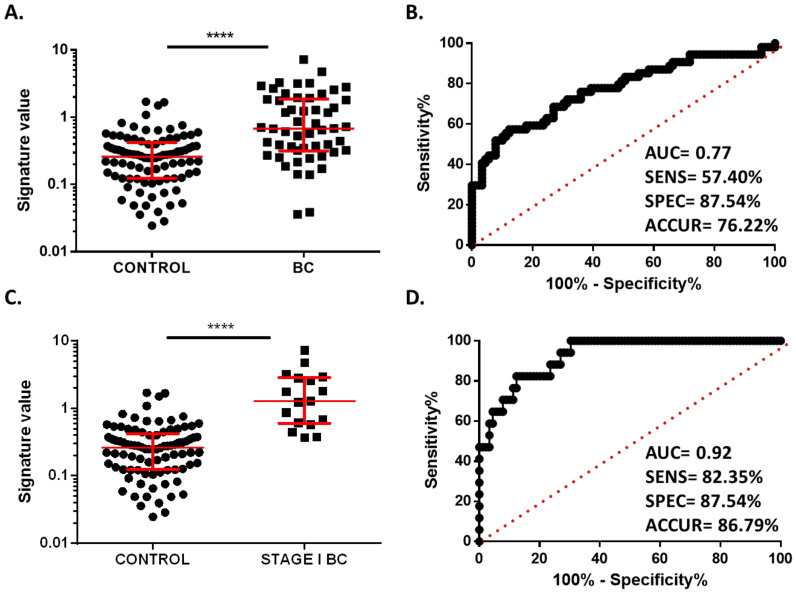
Diagnostic value of miR-30b-5p and miR-99a-5p combined signature. (**A**) Signature value of plasma samples of 54 BC patients and 89 healthy controls from discovery cohort. (**B**) Receiver operating characteristics (ROC) curve analysis of combined signature in plasma samples from discovery cohort. (**C**) Signature value of plasma samples of 17 stage I BC patients and 89 healthy controls from discovery cohort. (**D**) ROC curve analysis for combined signature in stage I BC patients. Red lines represent median ± interquartile range. **** *p* < 0.0001.

To evaluate the ability of these two miRNAs to distinguish BC patients from healthy controls, we created a combined plasma miRNA signature, and ROC analysis was performed. As expected, the miRNA signature value was higher in plasma from BC patients (median 1.230, 95% CI 0.3943–1.234) than in plasma samples from healthy controls (median 0.3319, 95% CI 0.2646–0.3991) (*p* < 0.0001) (Figure 3A). In addition, the combined signature was able to discriminate between BC patients and controls with an AUC of 0.77 (95% CI 0.6856–0.8538; *p* < 0.0001), and biomarker parameters of 57.4% sensitivity, 87.54% specificity, and 76.22% accuracy were obtained (Figure 3B).

Next, we analyzed the potential of this signature to identify BC at the very initial stage of the disease. For this purpose, we selected the 17 stage I BC patients included in the discovery cohort. The signature value was statistically higher in stage I BC patients (median 1.969, 95% CI 0.6117–2.809) than in healthy donors (median 0.3319, 95% CI 0.2073–0.3098) (*p* < 0.0001). ROC analysis showed an AUC of 0.9273 (95% CI 0.8714–0.9832, *p* < 0.0001) and biomarker performance parameters of 82.35% sensitivity, 87.54% specificity, and 86.79% accuracy (Figure 3C,D). These results demonstrate that the two-miRNA signature has strong potential as a diagnostic plasma biomarker in the very initial stages of BC.

**Figure 4:**
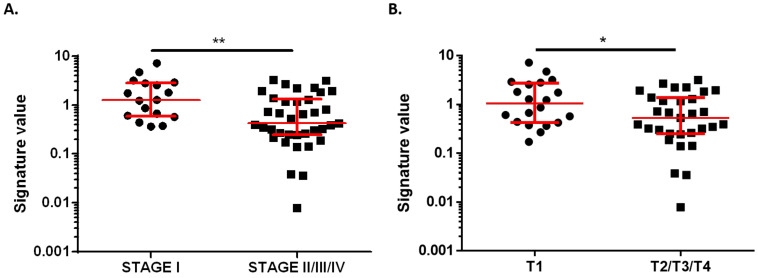
Association between combined signature value and clinicopathological data. (**A**) Signature value in plasma samples from very early-stage BC patients (stage I, *n* = 17) and in patients with advanced disease (stage II, III, IV, *n* = 37). (**B**) Signature value in plasma samples from BC patients with tumor size ≤ 20 mm (T1, *n* = 20) and in patients with tumor size >20 mm (T2, T3, T4, *n* = 31). Red lines represent median ± interquartile range. * *p* < 0.05; ** p < 0.01.

Then, comparisons between miRNA signature and clinicopathological features from all BC patients were carried out. Correlation with histological subtype, histological grade, metastasis, ki67, axillary lymph node affection, stage, and tumor size was evaluated. There was no association between signature value and histological subtype, histological grade, metastasis, ki67, and axillary lymph node affection. However, plasma samples from stage I BC patients showed a higher signature value (median 1.287, 95% CI 0.6117–2.809) than patients with more advanced disease (stages II, III, and IV, median 0.4249, 95% CI 0.3024–0.8092) (*p* = 0.005) (Figure 4A). According to previous data, patients with tumors classified as T1 (size ≤ 20 mm) showed a significantly higher value of miRNA signature (median 1.049, 95% CI 0.4424–2.566) compared to samples from patients presenting T2, T3 or T4 tumors (size > 20 mm, median 0.5300, 95% CI 0.3024–1.177) (*p* = 0.0235) (Figure 4B). These results are in line with the better biomarker diagnostic potential observed for stage I BC patients when compared to all BC stages. 

### 3.4. Validation of Plasma miRNA Signature as an Early Diagnosis BC Biomarker

**Figure 5:**
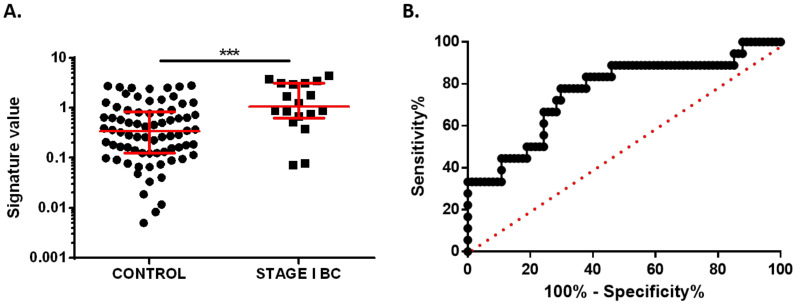
Diagnostic value of miR-30b-5p and miR-99a-5p combined signature as a very early BC diagnostic biomarker in validation cohort. (**A**) Signature value in 18 stage I BC patients and 74 healthy controls. (**B**) ROC curve analysis for combined signature in stage I BC patients. Red lines represent median ± interquartile range. *** *p* < 0.001.

To further validate our previous results, we selected a validation cohort from an independent institution. A total of 74 BC patients and 74 healthy volunteers’ samples were sequentially collected and all stage I BC samples (*n* = 18) were selected. Clinicopathological data from the validation cohort are detailed in Table 2. The early diagnostic potential of the proposed combined signature was blindly validated. As expected, the signature value was statistically higher in stage I BC patients (median 1.064, 95% CI 0.6722–3.102) than in healthy controls (median 0.3381, 95% CI 0.2076–0.5079) (*p* = 0.0004) (Figure 5A). ROC analysis demonstrated the ability of the signature to identify stage I BC patients in this independent cohort with an AUC of 0.7620 (95% CI 0.6305–0.8935). The biomarker performance parameters obtained with the optimal cut-off value from the discovery cohort reached 77.77% sensitivity, 68.91% specificity, and 70.65% accuracy (Figure 5B).

### 3.5. Functional Enrichment Analysis for miR-30b-5p and miR-99a-5p

**Figure 6:**
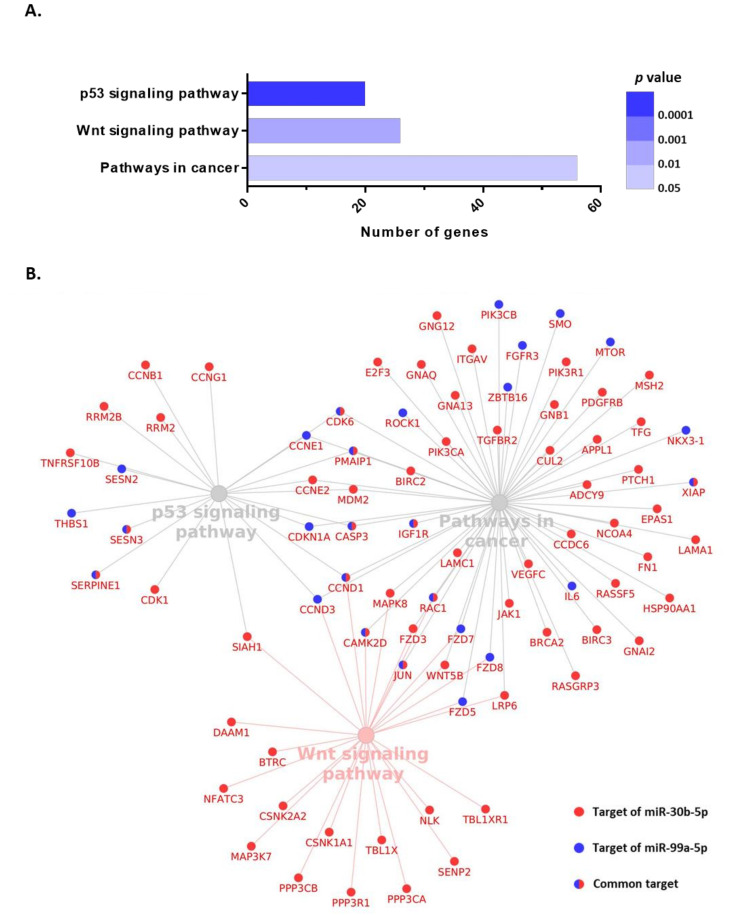
KEGG pathway enrichment analysis of miR-30b-5p and miR-99a-5p using DIANA Tools MirPath v3.0. (**A**) Significantly enriched pathways. (**B**) Interaction between miRNAs and their predicted target genes.

To further explore the association of these circulating miRNAs and clinical behavior, functional enrichment analysis based on KEGG annotation allowed for the identification of the pathways simultaneously targeted by miR-30b-5p and miR-99a-5p. Three pathways surfaced as simultaneously regulated by these two miRNAs: p53 signaling pathway, Wnt signaling pathway, and pathways in cancer (Figure 6A). Importantly, all these three pathways are cancer-related and strongly associated with tumor initiation. The p53 signaling pathway accounted for 20 targeted genes, the Wnt signaling pathway included 26 targeted genes, and pathways in cancer contained 56 targeted genes. From a total of 83 targeted genes, 10 were simultaneously targeted by miR-30b-5p and miR-99a-5p. All targeted genes and their interactions are shown in Figure 6B.

## 4. Discussion

The detection of BC in the initial stages allows for increased long-term survival rates. In this context, mammography is the main approach for BC screening, regardless of its limitations [4]. However, around 15% of patients are diagnosed at stage III or IV—locally advanced and metastatic stages of BC and related to worse prognosis [3]. In this scenario, new tools for early BC detection are needed to increase the number of patients diagnosed at early stages, thus improving prognosis and general survival rates.

Recent studies proposed liquid biopsy as a valuable procedure for cancer detection that might complement current clinical tools, improving not only diagnosis but also treatment monitoring [28]. The most studied biomarkers present in the bloodstream by cancer cells are circulating tumor DNA (ctDNA), circulating tumor cells (CTCs), circulating miRNAs, extracellular vesicles (EVs), and exosomes [29]. On one hand, ctDNA analysis is well studied in several cancers and it has been approved by the Food and Drug Administration (FDA) for treatment sensitivity determination in non-small cell lung cancer [30]. Together with ctDNA, CTCs have also been approved by the FDA as biomarkers for prognosis determination in colorectal, breast, and prostate cancer [31,32,33].

On the other hand, circulating miRNAs and EVs are still in development as non-invasive biomarkers. miRNAs have high stability in body fluids and have been demonstrated to be deregulated in most cancer types even at initial stages. Due to these facts, the detection of circulating miRNAs has emerged as a promising liquid biopsy strategy. Thus, several studies have evaluated circulating miRNA expression levels in different types of cancer as prognostic, predictive, diagnostic, and disease monitoring biomarkers [34]. 

Particularly, circulating miRNAs are of major interest in cancer detection as they have been proved to be released by malignant cells and to be differentially expressed in body fluids from healthy donors and cancer patients. Nowadays, circulating miRNAs in plasma have been demonstrated to have the potential to detect cancer even in the earliest stage of the disease [35]. In this context, a single miRNA as a diagnostic biomarker has some limitations regarding specificity and sensitivity. Due to this fact, it has been suggested that miRNA panels or signatures would increase the quality of proposed diagnosis biomarkers. Indeed, several plasma miRNA signatures have been proposed for diagnosis in different types of cancer such as bladder [36], cervical [37], colon [38,39,40], gastric [41,42], pancreas [43], osteosarcoma [44], oral [45], nasopharyngeal [46] and lung cancer [47]. Particularly, several studies proposed plasma miRNA signatures for BC diagnosis. Shen et al. [48] validated a combination of miR-148b and miR-133a. Mir-148b also takes part in a three-miRNA signature studied by Cuk et al. together with miR-409-3p and miR-801 [49], while miR-148a, miR-23a-3p, miR-130a-5p, miR-144-3p, and miR-152-3p combination was validated by Li et al. [50]. Frerès et al. proposed an eight-miRNA signature composed of miR-16, let-7d, miR-103, miR-107, miR-148a, let-7i, miR-19b, and miR-22* as a BC screening tool [51]. Additionally, Li et al. evaluated all members of the miR-106a–363 cluster and proposed a four-miRNA panel for BC detection [52]. In addition, Fang et al. described a panel composed of miR-30a-5p, miR-382-5p, miR-192-5p, miR-574-5p, miR-21-3p, and miR-221-3p, which has the potential to distinguish BC not only from healthy tissue but also from benign lesions [53].

Herein, we propose a two-miRNA signature based on the expression of miR-30b-5p and miR-99a in plasma samples as an early-stage BC detection biomarker. Our data show the potential value of this signature in distinguishing particularly stage I BC without any clinical manifestation from healthy samples. ROC analysis in the testing cohort of 106 individuals presented an AUC of 0.92 and 82.35% sensitivity, 87.54% specificity, and 86.79% accuracy. Moreover, we blindly validated our proposed signature in a second cohort of 92 individuals from an independent institution and our results demonstrated the value of the signature as a diagnostic biomarker with an AUC of 0.76, 77.77% sensitivity, 68.91% specificity, and 70.65% accuracy. Given the importance of an early diagnosis to improve prognosis and survival rates, we must highlight the higher performance achieved by our signature in stage I BC compared to all BC patients. In addition, this non-invasive biomarker could be detected by an efficient, fast, and non-expensive liquid biopsy method in the very initial stage of the disease when patients could still be asymptomatic.

To further elucidate the biological relevance of these circulating miRNAs, we also analyzed the pathways commonly targeted by both miR-30b and miR-99a. Interestingly, our in silico analysis identified three significantly targeted pathways: p53 signaling pathway, Wnt signaling pathway, and pathways in cancer, which have been widely implicated in breast carcinogenesis, as well as other types of cancer. The p53 pathway is one of the most important dysregulated pathways in cancer and it governs a complex anti-proliferative transcriptional program. *TP53*, coding for the protein p53, is the most frequently mutated gene in cancer and it is linked to hereditary disease [54]. p53 cascade is activated in the presence of several stimuli such as DNA damage or metabolic deprivation, thus promoting cell-cycle arrest and apoptosis, which are key to prevent cancer by avoiding the accumulation of oncogenic mutations, and its dysfunction promotes cancer development by allowing mutated cells to proliferate [55]. The loss of p53 accelerates the oncogenic transformation process, metastatic colonization, and drug resistance [56]. Particularly in BC, *TP53* is the second most frequently mutated gene after *PI3KCA* and it is decisive in early events of the development of BC and in the process of advanced disease. These characteristics make TP53 mutations a prognostic and predictive biomarker in BC and also a potential target for new treatments [57,58].

The Wnt pathway is a key cascade in breast oncogenesis due to its implication in mammary gland development and morphogenesis. Wnt/β-catenin pathway mutations are rare in BC. However, hyperactivated signaling is commonly found and associated with worse prognosis, particularly in triple-negative BC. The epigenetic inactivation of Wnt pathway antagonists, such as WIF1, which is reduced in 60% of BC, is also frequent [59]. Wnt cascade has been demonstrated to be involved in stem cell maintenance, antitumor immune attack, cancer metabolism, and drug resistance in BC [60,61,62]. Although the Wnt pathway supposes a promising therapeutic target, currently, only a few drugs that inhibit Wnt signaling have entered into clinical trials for BC, and their effectiveness is still controversial [62,63].

Altogether, our results obtained in the discovery cohort and validated in a second set of plasma samples support the value of this two-miRNA signature for detecting early-stage BC. Future evaluation of this signature in a bigger and multicenter study is warranted. The good performance of this panel makes it a good option to complement the standard BC screening techniques in a cost-effective manner. The evaluation of miRNA signatures in plasma would be a fast, reproducible, and effective test to improve the percentage of patients diagnosed with stage I BC, thus achieving better survival rates and prognosis for BC patients. Nonetheless, an important limitation of circulating miRNA signatures is the variability observed between detection methods such as RT-qPCR, microarrays, and deep sequencing and also between body fluids such as serum and plasma. Thus, the establishment of an accurate and reliable circulating miRNA signature for BC detection is still in development. These challenges indicate the need for the validation and standardization of miRNA assessment in liquid biopsy before the translation to clinical practice takes place. 

## 5. Conclusions

In the present study, the proposed two-miRNA-based signature was demonstrated to have high potential as a non-invasive biomarker for the early diagnosis of BC, essential for prognosis improvement. Although we were able to confirm the high diagnostic accuracy in two independent cohorts of BC patients, larger multi-institutional studies are needed to confirm its applicability in clinical practice.

## Figures and Tables

**Table 1:** Clinicopathological characteristics of BC patients and healthy controls from Cohort #1.

Characteristics	N (%)	Median (95% CI)	*p* Value (vs. Control)	*p* Value
Healthy controls	89	0.6787 (0.3943–1.234)		
BC patients	54	0.2593 (0.2073–0.3098)	<0.0001	
Molecular subtype				
Luminal	32 (59.26%)	0.7656 (0.3228–1.383)	<0.0001	0.2750
TNBC	10 (18.52%)	0.4252 (0.1722–1.177)	0.02	
HER2	12 (22.22%)	1.233 (0.4249–2.229)	<0.0001	
Grade				
1	2 (3.70%)	0.3232 (0.2711–0.3753)	0.9697	0.4094
2	24 (44.44%)	0.8368 (0.3654–1.954)	<0.0001	
3	25 (46.30%)	0.4424 (0.3024–1.234)	<0.0001	
Not available	3 (5.56%)			
Stage				
I	17 (31.48%)	1.287 (0.6117–2.809)	<0.0001	0.0523
II	15 (27.78%)	0.3205 (0.1722–1.8498	<0.0001	
III	11 (20.37%)	0.4542 (0.2155–1.383)	0.0026	
IV	12 (22.22%)	0.7656 (0.3475–1.934)	<0.0001	
T				
T1	20 (37.04%)	1.049 (0.4424–2.566)	<0.0001	0.2170
T2	15 (27.78%)	0.6770 (0.3024–1.954)	<0.0001	
T3	11 (20.37%)	0.2620 (0.0387–1.934)	0.0089	
T4	5 (9.26%)	0.7220 (0.3475–3.180)	<0.0001	
Not available	3 (5.55%)			
N				
Positive	26 (48.15%)	0.4774 (0.3024–1.288)	<0.0001	0.0584
Negative	26 (48.15%)	1.021 (0.4424–2.566)	<0.0001	
Not available	2 (3.70%)			
Metastasis				
Yes	11 (20.37%)	0.7656 (0.3475–1.934)	<0.0001	0.5168
No	41 (75.93%)	0.6612 (0.3753–1.287)	<0.0001	
Not available	2 (3.70%)			

**Table 2:** Clinicopathological characteristics of stage I BC patients and healthy controls from Cohort #2.

Characteristics	BC Patients	Healthy Controls
Number	18	74
Median age in years (range)	54 (34–69)	55 (32–90)
Molecular subtype, N (%)		
Luminal	11 (61.11%)	
TNBC	3 (16.67%)	
HER2	3 (16.67%)	
Not available		1 (5.55%)
Grade, N (%)		
1	5 (27.78%)	
2	9 (50%)	
3	4 (22.22%)	
Stage, N (%)		
I	18 (100%)	
T, N (%)		
T1	18 (100%)	
N, N (%)		
Positive	18 (100%)	
Metastasis, N (%)		
No	18 (100%)	

## Data Availability

The data presented in this study are available on request from the corresponding author. The data are not publicly available due to ethical restrictions.
